# Editorial

**Published:** 1842-02-19

**Authors:** 


### THE MEDICAL EXAMINER.

### PHILADELPHIA, FEBRUARY 19, 1842.

Our cotemporaries of the medical press have almost universally dealt very fairly by this journal in crediting it for original matter extracted from its columns. Occasional failures to do us this trifling justice we are disposed to refer to accidental causes. We do not deem it amiss, however, to ask of the N. Y. Lancet the favour to credit us in future for the hospital reports which it transfers to its pages from the Examiner; and we suppose that our cotemporary, the American Journal of the Medical Sciences, will expect a similar favour. While on the subject, we may express the opinion that credit is due not only for the original articles which appear in a journal, but also for the selected matter, which is, in general, prepared at little less cost of time than the original. Our own rule is always to name the source whence our matter is taken. We sometimes, however, see pieces credited directly to a French or German periodical, which have passed through two or three different channels before reaching their *ultima thule.* The *Zeitschrift* or *Gazette*, a far removed claimant, receives all the honours of paternity, and the journal which has collated, and, perhaps, condensed and translated, is passed over without mention. This is but scant justice.

